# Surgical Outcomes of Instrumented and Uninstrumented Arthrodesis for Lumbar Spinal Stenosis: A Comparative Radiographic and Clinical Study

**DOI:** 10.2174/0115734056415024251126104233

**Published:** 2026-07-14

**Authors:** Carla-Lisette Garcia-Ramos, Julio-Roberto Perez-Oliva, Carlos-Alberto Carreon-Cerda, Diana-Laura Hernandez-Moctezuma, Ernesto Roldan-Valadez, Baron Zarate-Kalfopulos, Alejandro Reyes-Sanchez

**Affiliations:** 1 Instituto Nacional de Rehabilitación “Luis Guillermo Ibarra Ibarra”, Cirugia de Columna, 14389, Mexico City, Mexico; 2 Instituto Nacional de Rehabilitación “Luis Guillermo Ibarra Ibarra”, Division of Research, 14389, Mexico City, Mexico; 3 Department of Radiology, I.M. Sechenov First Moscow State Medical University (Sechenov University), 119992, Moscow, Russia

**Keywords:** Lumbar spinal stenosis, Instrumented arthrodesis, Uninstrumented arthrodesis, Decompression surgery, Sagittal balance, Lumbar lordosis, Clinical outcomes, Radiographic outcomes, Fusion rates, Surgical complications

## Abstract

**Introduction:**

Lumbar spinal stenosis (LSS) is a prevalent degenerative condition that severely impairs mobility and quality of life in older adults. Decompressive surgery, often combined with spinal arthrodesis, remains the cornerstone of treatment. However, the role of instrumentation, particularly the use of interbody cages, remains debated. Instrumented arthrodesis may enhance fusion and segmental stability but is associated with higher morbidity, while uninstrumented techniques are less invasive but may compromise long-term fusion. This study compared clinical, radiographic, and surgical outcomes of three approaches: decompression with instrumented arthrodesis using interbody cages, decompression with instrumented arthrodesis without cages, and decompression with uninstrumented arthrodesis.

**Materials and Methods:**

In this prospective comparative study, 74 patients with LSS underwent one of the three procedures at a spine-specialized center, with ≥24 months of follow-up. Patients were stratified into Group 1 (n = 30), instrumented arthrodesis with interbody cages; Group 2 (n = 20), instrumented arthrodesis without cages; and Group 3 (n = 24), uninstrumented arthrodesis. Clinical outcomes were assessed with the Oswestry Disability Index (ODI), Roland-Morris Disability Questionnaire (RMDQ), Short Form-36 (SF-36), and Visual Analog Scale (VAS) for lumbar and radicular pain. Radiographic assessments included sagittal balance, lumbar lordosis, and fusion, evaluated by two independent radiologists. Statistical analyses employed chi-square tests, paired and unpaired t-tests, Pearson correlations, and logistic regression.

**Results:**

All groups showed significant improvement in ODI, RMDQ, SF-36, and VAS scores (*p* < 0.001). Postoperative sagittal balance improved significantly in Groups 2 and 3 (*p* = 0.001), whereas changes in lumbar lordosis were not significant (*p* = 0.663). Fusion rates were highest in Group 2 (95%) and lowest in Group 3 (75%; *p* = 0.018). Complications were most frequent in Group 3 (20.8%), largely incidental durotomies and wound infections. Reoperations were most common in Group 1 (23%), predominantly due to adjacent segment disease and implant failure.

**Discussion:**

Despite comparable functional improvements, the three strategies demonstrated distinct radiographic and surgical profiles. Instrumented arthrodesis without cages achieved superior fusion and sagittal correction with fewer complications, whereas uninstrumented arthrodesis, though effective for symptom relief, carried higher intraoperative complication rates. Cage-assisted fusion did not improve clinical or radiographic outcomes and was associated with more reoperations, particularly due to adjacent segment disease. These findings support tailoring instrumentation to patient comorbidities, stability requirements, and the need for anterior column support.

**Conclusion:**

All three surgical techniques improved function and pain in LSS patients. Instrumented arthrodesis without interbody cages provided the most favorable balance between fusion success and complication risk. Cages should be reserved for selected patients requiring sagittal realignment or foraminal restoration. Given the increased reoperation burden associated with instrumentation, surgical planning should be individualized, prioritizing stability, complication avoidance, and patient-specific risk factors, such as bone quality and comorbidities.

## 1 INTRODUCTION

Lumbar spinal stenosis (LSS) is a leading cause of pain and disability among the elderly, often resulting in neurogenic claudication and reduced mobility [1]. It is primarily caused by degenerative changes, including facet joint hypertrophy, thickening of the ligamentum flavum, and intervertebral disc degeneration, which collectively narrow the spinal canal [2]. With increasing life expectancy, the global prevalence of LSS continues to rise, reinforcing its significance as a target of surgical intervention and clinical research [3].

Surgical decompression remains the primary treatment to relieve neural compression in symptomatic LSS patients. However, the decision to add spinal arthrodesis, particularly instrumented fusion, remains debated [4, 5]. While instrumented arthrodesis may improve spinal stability and radiographic fusion rates, it also increases surgical morbidity, costs, and the risk of adjacent segment degeneration [6]. Conversely, uninstrumented fusion offers a less invasive alternative but raises concerns regarding long-term stability and fusion integrity [7].

Notably, global variations in practice patterns have emerged. Scandinavian countries often favor uninstrumented arthrodesis to reduce surgical stress and postoperative complications, especially in elderly patients [6]. In contrast, many centers in North America widely adopt instrumented fusion techniques, including the use of pedicle screws and interbody cages, to optimize biomechanical outcomes [7]. These differences underscore the need for evidence-based comparisons of surgical approaches in LSS [8].

Radiographic evaluation plays a critical role in surgical planning and outcome assessment in LSS. Parameters, such as sagittal balance, lumbar lordosis, and fusion integrity, are essential for guiding operative decisions and monitoring postoperative recovery. However, few studies directly compare these radiographic outcomes across different fusion techniques, particularly in conjunction with long-term clinical outcomes.

The aim of this study is to evaluate and compare three surgical strategies for LSS-decompression with instrumented arthrodesis using interbody cages, decompression with instrumented arthrodesis without interbody cages, and decompression with uninstrumented arthrodesis-in terms of radiographic alignment, fusion success, complication and reoperation rates, and validated clinical outcomes. By integrating radiologic and clinical perspectives, our findings aim to guide individualized surgical decision-making for patients with lumbar spinal stenosis.

## 2 METHODS

### 2.1 Study Design

This was a prospective comparative study conducted at a single tertiary-care hospital specializing in spine surgery. The sample size was determined based on patient availability during the enrollment period and expected effect sizes derived from previous studies evaluating ODI and fusion success in similar populations. A formal a priori power calculation was not conducted. However, we performed a retrospective post hoc power analysis using the observed effect sizes for our primary outcomes. This analysis indicated that the study had >80% power to detect between-group differences in fusion rates but considerably lower power (<50%) for detecting differences in reoperation rates and subgroup analyses. These limitations must be taken into account when interpreting secondary outcomes; the final cohort size was sufficient to detect statistically significant differences between groups for primary clinical and radiographic outcomes (α = 0.05).

The study had a minimum follow-up period of 24 months. No patients were lost to follow-up within the 24-month period. Patients with incomplete outcome data were excluded prior to statistical analysis. The study was approved by the institutional ethics committee (protocol 88/22 AE-2023-1). All participants provided informed consent before enrollment.

### 2.2 Patient Population

Eligible patients were aged 40 years or older and had symptomatic lumbar spinal stenosis (LSS) with advanced disc degeneration (grades 7 or 8, modified Pfirrmann scale) at the affected levels. All patients had failed at least six months of conservative treatment. Surgical candidacy was confirmed by a multidisciplinary team, including spine surgeons, neurologists, and radiologists.

#### 2.2.1 **Exclusion Criteria** Included

- Significant coronal plane deformities or scoliosis
- History of prior lumbar spinal surgery
- Incomplete clinical or imaging documentation

This was not a matched-cohort study. All patients were prospectively enrolled and assigned to surgical groups based on the surgeon’s intraoperative strategy and clinical judgment rather than matching criteria.

### 2.3 Surgical Interventions

Patients were stratified into three groups based on surgical approach:

- **Group 1 (n = 30):** Decompression with instrumented arthrodesis using interbody cages
- **Group 2 (n = 20):** Decompression with instrumented arthrodesis without interbody cages
- **Group 3 (n = 24):** Decompression with uninstrumented arthrodesis

All surgeries were performed by experienced board-certified spine surgeons (>10 years of surgical practice), using standardized protocols to ensure procedural consistency.

### 2.4 Data Collection and Outcome Measures

#### 2.4.1 Demographic and Intraoperative Data

Variables recorded included patient age, sex, body mass index (BMI), comorbidities (*e.g.*, hypertension, diabetes, smoking status), number of treated levels, surgical duration, and intraoperative blood loss.

### 2.5 Clinical Outcomes

Functional and pain-related metrics were evaluated preoperatively and at 24-month follow-up using:

- Oswestry Disability Index (ODI)
- Roland Morris Disability Questionnaire (RMDQ)
- Short Form-36 (SF-36) - physical and mental components
- Visual Analog Scale (VAS) - lumbar and radicular pain

### 2.6 Radiographic Outcomes

#### 2.6.1 Radiographic Assessment Iincluded

- Sagittal balance, measured by sagittal vertical axis (SVA)
- Lumbar lordosis, measured *via* Cobb angle
- Fusion success, defined by the presence of continuous bony trabeculation and absence of motion on dynamic lateral radiographs

All imaging assessments were performed independently by two musculoskeletal radiologists, blinded to surgical technique. Interobserver reliability was excellent (Cohen’s kappa κ = 0.86).

### 2.7 Complications and Reoperations

Recorded complications included incidental durotomy, wound infections, and implant-related events. Reoperations were defined as any secondary spinal procedure performed within the follow-up period.

### 2.8 Statistical Analysis

Data were analyzed using IBM SPSS Statistics v27.0.1.0. All analyses were conducted using complete case data. Variables with missing values were excluded from individual analyses without imputation. Given that missing values represented <5% of the dataset (n=3 patients), complete-case (listwise deletion) analysis was performed, which is considered acceptable when missingness is minimal and plausibly random (Lee *et al*. 2021, TARMOS framework) [9].

The number of patients included in each test is specified in the corresponding tables. Normality was tested with the Kolmogorov-Smirnov test. For between-group comparisons, the chi-square test (categorical data) and independent t-tests (continuous data) were carried out. To address the potential impact of unequal group sizes, we repeated analyses using statistical tests that are robust to variance imbalance, including Welch’s t-test for continuous variables and Fisher’s exact test for categorical variables. These sensitivity checks confirmed that the main results remained in line with those obtained from the original analyses. Paired t-tests were used for pre-post comparisons. Correlations were examined using Pearson’s r. Subgroup analyses by age, sex, and number of fused levels were not performed due to limited sample sizes within individual strata. No interaction terms were included in the regression models. Binary logistic regression identified predictors of fusion success and complications. Future studies with larger samples may allow for sensitivity testing of modeling assumptions. A *p*-value < 0.05 was considered statistically significant.

### 2.9 Software and Imaging Tools

Data tabulation and figure generation were conducted using *Microsoft Excel v16.33* and *Adobe Photoshop CC v19.1.9*. Radiographic measurements were performed using institutional PACS software with calibrated angle and distance tools.

## 3 RESULTS

### 3.1 Patient Demographics and Baseline Characteristics

Of the 81 eligible patients initially assessed, 7 were excluded: 3 declined participation and 4 had incomplete baseline imaging. The final study cohort included 74 patients. A flow diagram depicting patient inclusion, exclusion, and group assignment is provided in Fig. (**S1**). Seventy-four patients were enrolled and divided into three surgical groups:

- **Group 1 (n = 30):** Decompression with instrumented arthrodesis using interbody cages
- **Group 2 (n = 20):** Decompression with instrumented arthrodesis without interbody cages
- **Group 3 (n = 24):** Decompression with uninstrumented arthrodesis

There were no statistically significant differences between groups in baseline demographics, including age (*p* = 0.768), body mass index (*p* = 0.822), or sex distribution (*p* = 0.503). The number of operated segments was similar among the groups (*p* = 0.056). However, symptom duration differed significantly (*p* = 0.001), indicating variability in disease chronicity at the time of surgery (Table **1**). Data completeness was above 95% for all recorded variables. No variables had more than 2% missing data. Missing entries were due to unrecorded BMI (n=1) and unavailable SF-36 scores at follow-up (n=2).

### 3.2 Clinical Outcomes

All groups exhibited significant postoperative improvements in disability and pain scores at 24 months.

- The Oswestry Disability Index (ODI) improved from a preoperative mean of 54.22 ± 12.68 to 24.7 ± 13.26 (*p* = 0.001).
- The Roland Morris Disability Questionnaire (RMDQ) scores decreased significantly in all groups (*p* = 0.001).
- The Short Form-36 (SF-36) showed enhanced physical and mental health components postoperatively (*p* = 0.001 for both).
- Visual Analog Scale (VAS) pain scores also improved:
  - Lumbar pain: 6.85 ± 1.77 to 2.07 ± 1.89
  - Radicular pain: 6.53 ± 1.54 to 1.85 ± 1.76 (*p* = 0.001 for both)

These results, detailed in Table **2**, demonstrate that all three surgical techniques achieved comparable clinical improvements, with no statistically significant differences in final postoperative functional or pain scores between groups (*p* > 0.05).

### 3.3 Radiographic Outcomes

#### 3.3.1 Sagittal Balance

Preoperative sagittal vertical axis (SVA) averaged 27.62 ± 13.00 mm, which improved to 20.88 ± 10.75 mm postoperatively across all groups (*p* = 0.001).

- **Group 2** and **Group 3** showed the most pronounced improvements (*p* = 0.001).
- **Group 1** achieved a smaller, though still significant, change (*p* = 0.034).

These differences are summarized in Table **3** and visually supported by (Fig. **1A-D**), which shows improved postoperative coronal and sagittal alignment on panoramic radiographs.

#### 3.3.2 Lumbar Lordosis

No significant changes were observed in lumbar lordosis across all groups (preoperative: 49.32 ± 10.86°; postoperative: 50.01 ± 9.27°; *p* = 0.663), as presented in Table **3**. This suggests that sagittal correction was primarily related to global alignment rather than segmental angulation.

#### 3.3.3 Fusion Success

The fusion rate in Group 2 was 95% (95% CI, 85.5-100%), in Group 1 was 93.3% (95% CI, 84.2-100%), and in Group 3 was 75% (95% CI, 58.1-91.9%). The difference was statistically significant (*p* = 0.018). Fusion was evaluated on dynamic lateral radiographs by two independent radiologists, with a high interobserver agreement (κ = 0.86). Representative cases are illustrated in Fig. (**2A-D**), highlighting radiographic evidence of cage placement, transpedicular screw fixation, and bony continuity.

### 3.4 Complications

The overall complication rate was 12.2% (9/74 patients).

- **Group 3** had the highest rate (20.8%):
  - Four incidental durotomies (16.7%)
  - One deep wound infection (4.2%)
- **Group 1:** 10% (two implant-related complications; one CSF leak)
- **Group 2:** 5% (one superficial wound infection)

Comparative data from prior studies and our findings are detailed in Table **4**, which shows variability in complication rates across surgical strategies and centers. The higher complication rate in Group 3 may reflect reduced mechanical stabilization, increasing intraoperative neural manipulation.

### 3.5 Reoperations

- **Group 1** had the highest reoperation rate (23%):
  - Three cases of adjacent segment disease (ASD)
  - Two implant failures with pseudoarthrosis
- **Group 2:** 20% (instability and progressive foraminal stenosis)
- **Group 3:** 4.2% (one case of nonunion requiring revision surgery)

Reoperation data are summarized in Table **5**, and imaging features associated with degenerative progression and foraminal narrowing are shown in Fig. (**3A-D**) (MRI T2-weighted views). Notably, adjacent segment pathology and hardware complications contributed significantly to reintervention in Group 1.

### 3.6 Multivariate Analysis

Logistic regression revealed:

- Instrumented arthrodesis positively correlated with fusion success (r = 0.27, *p* = 0.019)
- Longer hospital stays were associated with higher reoperation rates (r = 0.370, *p* = 0.001)
- No significant correlation between perioperative complications and reoperation risk (r = 0.052, *p* = 0.663).

## 4 DISCUSSION

### 4.1 Principal Findings

This comparative study evaluated three surgical approaches for LSS: decompression with instrumented arthrodesis using interbody cages, decompression with instrumented arthrodesis without cages, and decompression with uninstrumented arthrodesis. While all three techniques produced significant improvements in clinical outcomes, including pain relief and disability scores (Table **2**), they differed in fusion rates, sagittal balance correction, complication profiles, and reoperation rates.

Instrumented arthrodesis without interbody cages (*Group 2*) yielded the highest fusion rate (95%) and the most substantial improvement in sagittal balance (Table **3**), without increasing complications (5%) or reoperations (20%). In contrast, uninstrumented arthrodesis (*Group 3*) had the lowest fusion rate (75%) and highest complication rate (20.8%, Table **4**), whereas instrumented arthrodesis with cages (*Group 1*) was associated with the highest reoperation rate (23%, Table ****5****), primarily due to adjacent segment disease (ASD) and implant-related failure.

### 4.2 Comparison with Existing Literature

#### 4.2.1 Clinical Outcomes and Functional Recovery

All techniques significantly reduced lumbar and radicular pain and improved physical and mental functioning, confirming findings from the studies by Försth *et al*. and Ghogawala *et al*. [1, 2]. Our results support the existing literature, suggesting that while instrumentation may enhance radiographic fusion, it does not necessarily improve clinical outcomes compared with uninstrumented techniques [4, 10]. The comparable clinical improvements across groups despite differing fusion rates highlight a frequent clinical-radiographic discordance. In elderly patients with lumbar spinal stenosis, symptom relief often derives primarily from decompression of neural elements, and high fusion rates may not be necessary to achieve meaningful functional recovery. Prior studies have similarly demonstrated that patient-reported outcomes do not always parallel radiographic fusion success in degenerative spinal conditions [1, 4]. While achieving fusion may still reduce late instability and reoperation risk, our findings support a more nuanced interpretation in which radiographic fusion is not always synonymous with clinical benefit, particularly in older or comorbid populations.

### 4.3 Radiographic Outcomes and Sagittal Balance

Radiographically, Groups 2 and 3 achieved better postoperative sagittal balance than Group 1 (Table **3**), a result in line with those of Austevoll *et al*. [4], who reported similar findings using decompression with fusion. Interestingly, despite the use of interbody cages in Group 1, lumbar lordosis did not significantly improve (*p* = 0.663), suggesting that cage use may not be essential for achieving sagittal realignment in mild to moderate LSS. One explanation for the limited sagittal balance correction in Group 1 relates to cage-specific and patient-related factors. The biomechanical advantage of interbody cages depends on proper positioning, lordotic angle, and preservation of endplate integrity. Suboptimal cage placement or subsidence can reduce the intended sagittal correction. Group 1 included patients with greater baseline sagittal imbalance, which constrained postoperative improvement. Surgeon preference for cage use in patients with more severe degeneration may have introduced selection bias. Recent reports confirm that sagittal realignment after lumbar fusion is more strongly influenced by cage geometry, positioning, and spinopelvic parameters than by cage presence alone [11, 12]. These factors likely explain why cage use did not yield superior correction of sagittal alignment in our cohort.

Radiologic improvements in sagittal vertical alignment (SVA), illustrated in Fig. (**1**), were most pronounced in Group 2, indicating that well-performed posterolateral fixation without cages may be sufficient for restoring balance. These findings align with recent reports emphasizing the role of alignment correction without over-reliance on cages [5, 13].

### 4.4 Fusion Rates and Radiologic Interpretation

A methodological strength of this study was the rigor of radiographic evaluation. All imaging assessments were performed independently by blinded reviewers, achieving high interobserver agreement (κ=0.86). This reliability reinforces the validity of our fusion rate comparisons and supports confidence in the radiographic interpretation of outcomes across groups.

Fusion was most successful in instrumented groups, particularly Group 2 (Fig. **2**), reinforcing previous studies demonstrating the benefit of posterior instrumentation in achieving bony union [1, 2, 14]. However, adding interbody cages did not significantly increase fusion rates compared to standard instrumentation, as supported by Robinson *et al*. [14].

### 4.5 Complications and Reoperations

The high complication rate in Group 3 (20.8%), including incidental durotomies, mirrors previous findings by Said *et al*. [15], Koenig *et al*. [16], and Jalapour *et al*. [17], highlighting that decompression without stabilization may increase neural manipulation risks. Group 1, despite its biomechanical advantages, had the highest reoperation rate (23%), driven primarily by adjacent segment degeneration (Table **5**), in line with Austevoll *et al*. and Weinstein *et al*. [4, 8]. In our cohort, the elevated complication rate for uninstrumented arthrodesis (28.8%) compared to instrumented formation (8%) may reflect the increased technical demands of decompression without mechanical stabilization, which can require more extensive neural manipulation and increase the risk of incidental durotomy or neural injury. Patients in the uninstrumented group were generally older and had a greater comorbid burden, factors known to predispose to postoperative complications, such as wound infection or delayed recovery. Variability in perioperative care, surgical technique, and institutional protocols may further explain the discrepancies with some prior studies. For example, Anaspure *et al*. [18] reported that while instrumented fusion achieved significantly higher fusion rates, complication and reoperation rates across instrumented *versus* non-instrumented fusion remain variable, suggesting that patient selection and technical factors significantly influence outcomes.

The differential complication patterns across groups also deserve attention. Group 3 showed a higher frequency of durotomies, likely attributable to increased neural manipulation during decompression without the stabilizing effect of instrumentation. In contrast, Group 1 demonstrated a greater incidence of adjacent segment disease, in line with the biomechanical consequences of rigid fixation, which can accelerate degenerative changes at adjacent motion segments. Similar findings have been reported in recent literature, with higher durotomy risk observed in uninstrumented procedures [19] and increased ASD incidence documented following instrumented fusion [20]. These data reinforce the notion that each surgical strategy carries distinct trade-offs that must be carefully weighed during decision-making.

Reoperation rates varied significantly across studies, with instrumented fusion in our study showing a rate of 12.0%, in contrast to the 1.9% reported by Andresen *et al*. [21] and Austevoll *et al*. [4]. In our cohort, the relatively high reoperation rate observed in the instrumented group (12.0%) was primarily attributable to adjacent segment disease and hardware-related complications, both of which are recognized long-term risks associated with rigid fixation. This finding contrasts with the lower reoperation rate in the uninstrumented group (8.3%), where reduced mechanical stress at adjacent levels may have contributed to fewer late revisions, despite lower fusion rates and higher intraoperative complication rates. The discrepancy with prior studies may also reflect differences in patient selection, baseline sagittal balance, and follow-up duration. Notably, Andresen *et al*. [21] reported significantly lower reoperation rates for instrumented fusion, while Kaiser *et al*. [10] found no consistent advantage between strategies, underscoring that reoperation risk is highly context-dependent. Our results suggest that while instrumentation can improve fusion integrity, it may increase long-term mechanical stress on adjacent segments, leading to higher revision rates. Meanwhile, Kaiser *et al*. [10] and Jalapour *et al*. [17] reported higher reoperation rates for uninstrumented arthrodesis, supporting the notion that this technique may lead to a greater risk of delayed instability.

The elevated reoperation rate observed in Group 2 (20%), despite high fusion success (93.3%), likely reflects the biomechanical trade-offs of posterior-only fixation without interbody support. Although fusion was achieved, the absence of cages may have limited restoration of disc and foraminal height, contributing to recurrent or progressive stenosis. Posterior constructs alone also concentrate mechanical stress at the pedicle screw-rod interface, predisposing to hardware-related complications. These mechanisms are in line with recent reports showing that cage geometry and positioning strongly influence disc and foraminal height restoration, with suboptimal restoration associated with recurrent stenosis [22]. Bone quality strongly influences construct durability, as highlighted by Lechtholz-Zey *et al*. [23], underscoring the role of host factors in the long-term stability of posterior-only instrumentation.

Conversely, instrumented fusion with interbody cages (Group 1) demonstrated the highest reoperation rate (23%), primarily due to adjacent segment disease (ASD) and implant-related complications (Table **5**). This aligns with evidence suggesting that instrumented fusion may increase the mechanical stress at adjacent spinal levels, leading to accelerated degeneration and a higher likelihood of reoperation (Austevoll *et al*.) [4].

MRI findings in Fig. (**3**) support the hypothesis that instability and foraminal narrowing are major contributors to postoperative deterioration and the need for reintervention. These radiologic correlates validate the predictive role of imaging in surgical outcome assessment.

### 4.6 Clinical Implications and Surgical Strategy

Our findings support a tailored surgical approach to LSS. Instrumented arthrodesis without interbody cages (Group 2) provided the most favorable balance of fusion success, sagittal alignment, and low complication rates. Interbody cages did not provide additional benefit for radiographic alignment or fusion success and were associated with higher reoperation rates. These results argue for selective rather than routine use of interbody devices, especially in elderly patients or those with limited functional demands.

For patients with stable sagittal profiles and no signs of instability, uninstrumented arthrodesis remains a viable option, offering comparable clinical improvement while reducing surgical complexity. However, the risk of lower fusion success and higher complication rates must be considered. A practical decision framework for selecting the fusion method in LSS is therefore warranted. In patients without radiographic or procedural instability and with limited facet resection anticipated, decompression alone or uninstrumented fusion may be reasonable, as contemporary randomized trials and systematic reviews have shown no consistent clinical advantage to routine fusion in all cases of degenerative stenosis or low-grade spondylolisthesis [1, 4, 10]. When dynamic or intraoperative instability is evident, when multilevel decompression with substantial facetectomy is planned, or when sagittal realignment is a goal, instrumented fusion is favored to maintain stability and reduce delayed instability-related reintervention [4, 21]. Bone health is integral to method selection: osteoporosis or low CT-derived Hounsfield units increase the risk of screw loosening, cage subsidence, and mechanical failure; these patients benefit from preoperative optimization and may require augmented fixation strategies or selective avoidance of interbody cages when endplate quality is poor [23-25]. Host factors also guide choice: smoking and advanced age increase pseudarthrosis risk; diabetes elevates infection and medical complication risks; these considerations weigh toward the least morbid construct that still achieves stability and symptom relief [26, 27]. Finally, durotomy risk rises with older age, revision status, and multilevel decompression; in such settings, minimizing neural manipulation and tailoring the extent of decompression may reduce complications regardless of fixation strategy [19, 20].

Cost-effectiveness and morbidity considerations are also essential when evaluating cage use. Although interbody cages may improve disc height and segmental lordosis, systematic reviews and comparative trials have shown that these radiographic improvements do not consistently yield superior patient-reported outcomes over posterolateral fusion alone [28]. Cages increase surgical costs and may introduce specific risks, such as subsidence or endplate violation, which can compromise long-term stability [29]. Recent cost-effectiveness analyses provide mixed signals. Yee *et al*. [30] found that interbody fusion can be cost-effective in selected high-risk subgroups, while Sastry *et al*. [31] reported that decompression alone was more cost-effective than decompression plus fusion over a two-year horizon. Overall, cage use should not be routine but reserved for cases requiring height restoration, foraminal decompression, or sagittal realignment; in our cohort, instrumentation without cages offered a favorable balance of fusion, alignment, and complication risk, while routine cage use provided no added benefit.

### 4.7 Contribution to Radiology and Imaging-based Decision-making

This study reinforces the central role of imaging, not only in preoperative planning but also in long-term surgical evaluation. Standardized radiologic metrics, such as sagittal balance, lumbar lordosis, and fusion assessment, guided objective comparisons across techniques. Radiographic correlations with clinical outcomes (Figs. **1**-**3**) enhance the interpretive value of follow-up imaging in spine surgery. These findings support the integration of imaging specialists into multidisciplinary surgical teams to improve patient-specific planning and prognosis prediction.

### 4.8 Limitations and Strengths

This study has several limitations. It was conducted at a single institution, potentially limiting external validity. Although the follow-up period (24 months) captured early and mid-term outcomes, longer-term degenerative changes, particularly adjacent segment pathology, may not have fully manifested. Another limitation of our study is the non-randomized nature of patient allocation. Group assignment was determined by the operating surgeon’s intraoperative judgment and clinical assessment, which may have introduced selection bias. This design increases the risk that unmeasured confounders, such as underlying disease severity, bone quality, or comorbid burden, may have influenced both the choice of surgical technique and the outcomes observed. For example, differences in symptom duration across groups suggest possible imbalances that may have impacted results. While our prospective design and standardized surgical protocols mitigate some of these concerns, the absence of randomization limits the ability to fully exclude allocation bias [21].

The absence of a formal sample size calculation represents a limitation. Unequal sample sizes across groups, particularly the smaller cohort in Group 2 (n=20), may have reduced the power to detect certain differences in complication and reoperation outcomes. Although sensitivity analyses using variance-robust statistical methods confirmed the robustness of our primary results, these imbalances represent a limitation that should be addressed in future studies with larger and more evenly distributed samples. Our post hoc power analysis confirmed adequate power to detect differences in fusion rates; however, the study was underpowered for reoperation outcomes and subgroup comparisons. As a result, these findings should be considered exploratory and warrant confirmation in larger randomized trials [4]. Another limitation concerns the management of missing data. Although complete-case analysis with <5% missingness is generally acceptable, we acknowledge that even such minimal exclusions may introduce bias under non-random missingness. We considered the TARMOS guidelines (Lee *et al*. 2021) in our reporting, which promote transparency and sensitivity to missing-data assumptions [9].

Our sample size limited the feasibility of performing stratified subgroup analyses by clinically important variables, such as age, body mass index, and comorbidities (*e.g.*, diabetes, smoking, osteoporosis). These factors are well-recognized as modifiers of fusion success, complication risk, and reoperation likelihood [23, 26, 27]. Another limitation relates to our multivariate analysis. Due to sample size constraints and event frequency, we restricted the number of covariates included in the logistic regression model. As a result, key predictors of fusion success and complications, such as BMI, smoking status, and bone quality, were not incorporated. Omitting these factors may have introduced residual confounding. Although we prospectively recorded these variables, dividing the cohort into subgroups would have resulted in underpowered analyses. Future studies with larger sample sizes should aim to integrate comprehensive predictors, including bone density metrics and lifestyle factors, which have been shown to significantly influence implant stability and fusion outcomes [23, 24]. Larger sample sizes should incorporate stratified analyses to identify patient-specific risk profiles that may influence surgical outcomes.

A strength of our study is the complete follow-up rate at 24 months. Full retention of all patients minimizes attrition bias and enhances the internal validity of our longitudinal comparisons. Another strength of our study is the multidimensional outcome assessment, which combined patient-reported measures (ODI, SF-36), radiographic evaluations (fusion and sagittal alignment), and surgical variables (complications and reoperations). This comprehensive design enhances the generalizability of our findings and ensures that both structural integrity and functional recovery were evaluated. The generalizability of our findings may be limited by the single-center design. Our institution is a high-volume tertiary referral center with experienced spine surgeons and standardized perioperative care, conditions that may not reflect all practice environments. In lower-volume or resource-limited settings, differences in surgical expertise, implant access, and perioperative support may influence fusion success, complication rates, and cost-effectiveness. Future multicenter studies across varied health systems are required to validate the external applicability of our results.

### 4.9 Future Directions

Future research should prioritize multicenter randomized controlled trials to strengthen the external validity of findings. Key areas include:

- **Long-term Outcomes (>5 years):** to better define the incidence and clinical impact of adjacent segment degeneration and hardware-related complications.
- **Selective use of Interbody Cages:** evaluating their value in specific high-risk subpopulations, such as smokers, osteoporotic patients, and those with advanced sagittal imbalance, where the balance between mechanical benefit and complication risk may differ.
- **Head-to-head Comparisons of Fusion Strategies:** randomized trials directly comparing instrumented arthrodesis without cages and uninstrumented fusion in vulnerable groups (*e.g.*, osteoporotic or elderly patients) to determine whether the benefits of instrumentation outweigh its costs and potential morbidity.
- **Integration of Advanced Imaging Biomarkers:** such as CT-derived Hounsfield units, MRI-based radiomics, or quantitative sagittal alignment parameters, for early detection of nonunion, screw loosening, or progressive sagittal imbalance, enabling individualized risk stratification.

## CONCLUSION

This prospective study compared three surgical techniques for LSS: decompression with instrumented arthrodesis using interbody cages, instrumented arthrodesis without cages, and uninstrumented arthrodesis. All approaches significantly improved clinical outcomes, including disability scores and pain relief, but differed in radiographic results, fusion success, complication profiles, and reoperation rates.

Instrumented arthrodesis without interbody cages (Group 2) achieved the highest fusion rate (95%) and the greatest correction in sagittal balance, with the lowest complication incidence. Uninstrumented arthrodesis (Group 3), while effective for symptom relief, showed lower fusion success (75%) and higher complication rates (20.8%). Instrumented arthrodesis with interbody cages (Group 1) was associated with the highest reoperation rate (23%), mainly due to adjacent segment degeneration and hardware failure.

Radiographic assessments (sagittal balance, lumbar lordosis, and fusion integrity) play a central role in comparing surgical strategies. These findings highlight the value of imaging in guiding treatment selection and postoperative monitoring.

Overall, instrumented fusion without interbody cages emerged as the most balanced option, offering radiographic stability with a favorable risk profile. Importantly, our results suggest that routine cage use is not warranted. Cages should be avoided in patients with a low risk of nonunion and without sagittal imbalance or foraminal collapse, while their use may be reserved for selected cases requiring anterior column support, foraminal height restoration, or sagittal realignment. This qualified approach strengthens individualized surgical planning by balancing efficacy, safety, and cost.

Surgical planning for LSS should remain individualized, taking into account patient-specific factors, such as mechanical stability, comorbidities, bone quality, and long-term risk tolerance. Imaging-guided decision-making continues to be essential for optimizing outcomes and minimizing complications.

Beyond clinical outcomes, our findings also resonate with Sustainable Development Goals (SDGs). Selective avoidance of unnecessary cage use reduces surgical costs while preserving patient benefit, aligning with SDG 3.8 (Universal Health Coverage). Tailoring fixation strategies to bone quality helps minimize implant-related failures, contributing to SDG 9 (Industry, Innovation, and Infrastructure) by promoting efficient use of medical technologies. The emphasis on individualized, evidence-based decision-making fosters knowledge transfer in surgical education, in line with SDG 4 (Quality Education).

## Figures and Tables

**Fig. (1):**
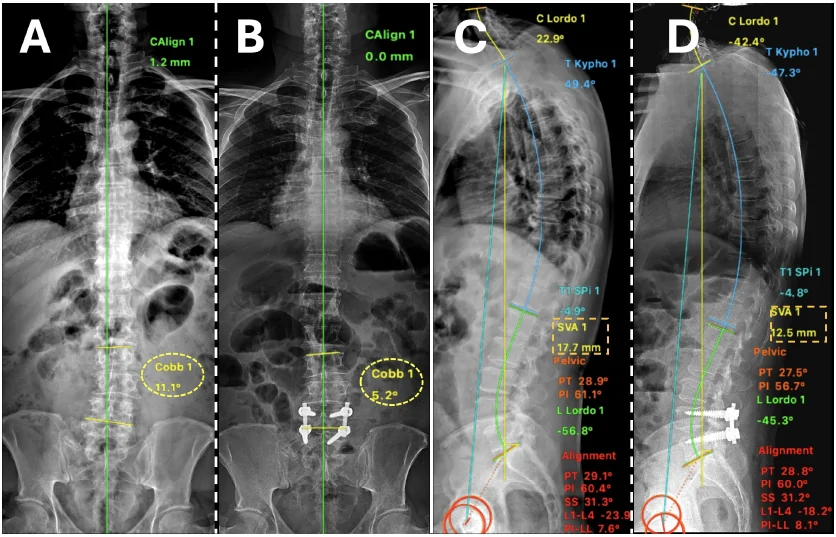
Preoperative and postoperative panoramic radiographs showing coronal and sagittal balance improvements. (**A** and **B**) Preoperative and postoperative panoramic anteroposterior radiographs were used to assess coronal balance. Postoperative images show an improved balance with a neutral sagittal alignment and a reduction of the L2-L4 Cobb angle from 11.1° to 5.2° (yellow dashed ellipses). (**C** and **D**) Preoperative and postoperative lateral panoramic radiographs, demonstrating postoperative improvement in sagittal balance (SVA) from 17.7 mm to 12.5 mm (orange dashed square). Following fixation, spinal-pelvic parameters and lumbar lordosis measurements indicate alignment within normal parameters.

**Fig. (2):**
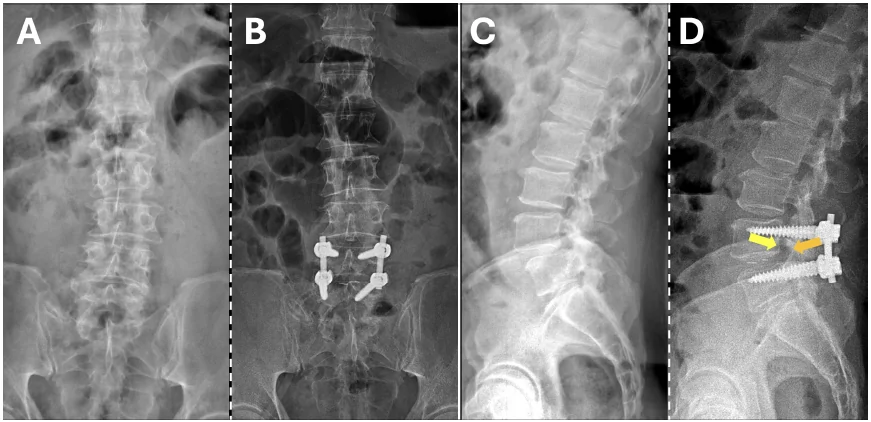
Preoperative and postoperative radiographic evaluation of a patient with lumbar spinal stenosis and spondylolisthesis. A 59-year-old male patient presenting with intermittent claudication, L4-L5 radicular symptoms, and chronic low back pain. The diagnosis includes L4-L5 lumbar spinal stenosis and Meyerding grade I L4-L5 spondylolisthesis. (**A** and **B**) Preoperative and postoperative anteroposterior lumbar radiographs. (**C** and **d**) Preoperative and postoperative lateral radiographs. The postoperative images show correction of the listhesis (yellow arrow) and expansion of the L4-L5 foraminal space (orange arrow). In both postoperative projections (**B** and **D**), proper fixation with lumbar transpedicular screws and placement of a PEEK (polyetheretherketone) interbody cage is observed. The procedure performed was a posterior lumbar approach with L4-L5 recalibration and 360° L4-L5 arthrodesis.

**Fig. (3):**
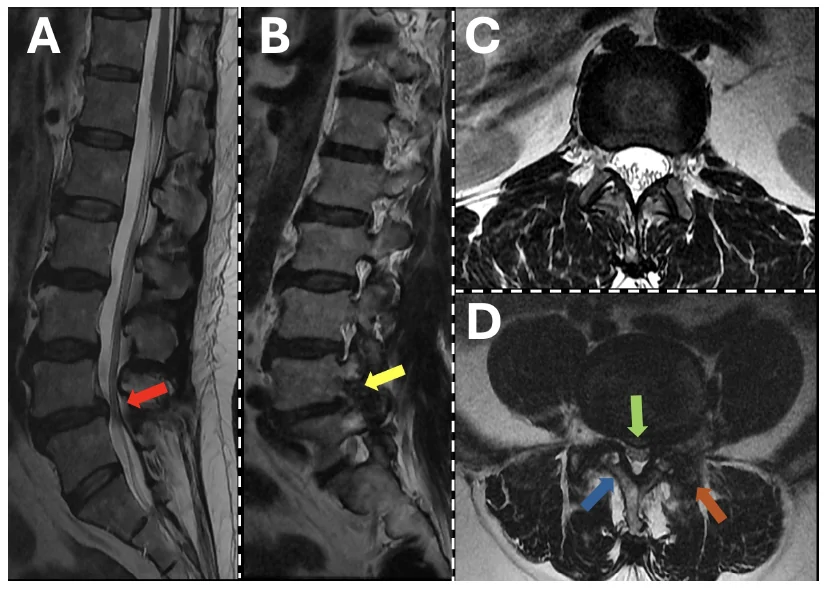
T2-weighted MRI findings demonstrating lumbar spinal stenosis and associated degenerative changes. (**a**) Central sagittal section revealing an L4-L5 disc herniation that reduces vertebral canal space and contacts the cauda equina nerve roots (red arrow). (**b**) Left parasagittal section showing near-complete occlusion of the L4-L5 foraminal space (yellow arrow). (**c**) Axial section at the L3-L4 level, demonstrating a normal lumbar canal without disc involvement or significant facet degeneration. The nerve roots are widely distributed within the vertebral canal. (**d**) Axial section at the L4-L5 level, revealing a central disc herniation (green arrow), global disc bulging, ligamentum flavum thickening (green arrow), and facet joint osteoarthritis (orange arrow). These three conditions contribute to reduced vertebral canal space, causing root crowding in the central canal.

**Table 1:** Baseline Characteristics of Patients Undergoing Instrumented and Uninstrumented Arthrodesis for Lumbar Spinal Stenosis. Table 1 presents the baseline demographic, clinical, and surgical characteristics of patients undergoing instrumented arthrodesis with interbody cages (Group 1), instrumented arthrodesis without interbody cages (Group 2), and uninstrumented arthrodesis (Group 3). No significant differences were observed in age (*p* = 0.768), BMI (*p* = 0.822), intraoperative blood loss (*p* = 0.198), or surgical time (*p* = 0.198) among the groups, indicating a comparable baseline profile. Symptom duration varied significantly between groups (*p* = 0.001), suggesting differences in the timing of surgical intervention. The percentage of male patients was similar across groups (*p* = 0.503), and the number of operated segments did not differ significantly (*p* = 0.056). These findings confirm that the study groups were well-matched for key preoperative variables, supporting the validity of comparative outcome analysis.

**Parameter**	**Group 1 (n=30)**	**Group 2 (n=20)**	**Group 3 (n=24)**	**Total (n=74)**	***p*-value**
**Age (years), M ± SD**	71.6 ± 6.66	69.60 ± 7.22	73.67 ± 5.91	71.73 ± 6.68	0.768
**Weight (kg), M ± SD**	70.03 ± 11.23	68.35 ± 8.07	69.92 ± 12.12	69.54 ± 10.67	0.131
**Height (m), M ± SD**	1.61 ± 0.09	1.60 ± 0.07	1.61 ± 0.11	1.61 ± 0.09	0.842
**BMI (kg/m^2^), M ± SD**	26.72 ± 72	26.62 ± 2.37	26.62 ± 3.37	26.66 ± 2.82	0.822
**Intraoperative blood loss (ml), M ± SD**	603.33 ± 404	452.50 ± 261.81	277.50 ± 197.44	456.89 ± 338.20	0.198
**Surgical time (hours), M ± SD**	3.63 ± 1.19	3.53 ± 0.93	2.50 ± 1.06	3.22 ± 1.15	0.198
**Male sex, % (n)**	50% (15)	50% (10)	54.2% (13)	51.4% (38)	0.503
**Comorbidities, M ± SD**	1.33 ± 1.24	0.90 ± 0.96	1.13 ± 1.07	1.15 ± 1.11	0.344
**Operated segments, M ± SD**	1.90 ± 0.80	2.10 ± 0.78	1.79 ± 0.88	1.92 ± 0.82	0.056
**Symptom duration (months), M ± SD**	6.48 ± 5.88	5.16 ± 4.08	6.92 ± 6.12	7.60 ± 5.52	0.001*

**Abbreviation:** M = Mean; SD = Standard Deviation; (n) = Number;

**Note:** *p < 0.05 indicates statistical significance.

**Table 2:** Comparison of Preoperative and Postoperative Clinical Outcomes Across Surgical Techniques for Lumbar Spinal Stenosis. Table **2** summarizes the preoperative and postoperative clinical outcomes across three surgical techniques: instrumented arthrodesis with interbody cages (Group 1), instrumented arthrodesis without interbody cages (Group 2), and uninstrumented arthrodesis (Group 3). All groups demonstrated significant postoperative improvements (*p* = 0.001) in functional disability (Oswestry Disability Index, ODI), physical function (Roland Morris Questionnaire), health-related quality of life (Short Form-36, SF-36), and pain intensity (Visual Analog Scale, VAS). Notably, ODI scores improved from 54.22 ± 12.68 preoperatively to 24.7 ± 13.26 postoperatively, and VAS lumbar pain scores decreased from 6.85 ± 1.77 to 2.07 ± 1.89. While no significant differences were observed between groups in terms of final functional and pain-related outcomes (*p* > 0.05), these results suggest that all three techniques effectively improve functional disability and pain relief in patients with lumbar spinal stenosis.

**Variable**	**Group 1 (n=30)**	**Group 2 (n=20)**	**Group 3 (n=24)**	**Total (n=74)**	***p*-value**
**ODI (Preoperative)**	57.43 ± 10.18	48.15 ± 6.10	55.25 ± 14.26	54.22 ± 12.68	0.591
**ODI (Postoperative)**	26.37 ± 11.82	22.35 ± 13.19	24.58 ± 15.16	24.7 ± 13.26	-
**ODI (Change, *p*-value)**	*p* = 0.001	*p* = 0.001	*p* = 0.001	*p* = 0.001	-
**Roland-Morris (Preoperative)**	17.07 ± 3.56	14.65 ± 5.31	16.38 ± 6.34	16.19 ± 5.13	0.187
**Roland-Morris (Postoperative)**	7.63 ± 4.19	6.15 ± 4.19	7.33 ± 5.13	7.14 ± 4.50	-
**Roland-Morris (Change, *p*-value)**	*p* = 0.001	*p* = 0.001	*p* = 0.001	*p* = 0.001	-
**SF-36 Physical (Preoperative)**	26.40 ± 7.85	30.15 ± 13.30	25.79 ± 8.37	27.22 ± 9.79	0.753
**SF-36 Physical (Postoperative)**	64.83 ± 16.31	73.25 ± 16.81	62.13 ± 21.26	66.23 ± 18.48	-
**SF-36 Physical (Change, *p*-value)**	*p* = 0.001	*p* = 0.001	*p* = 0.001	*p* = 0.001	-
**SF-36 Mental (Preoperative)**	42.10 ± 12.98	42.25 ± 13.98	43.04 ± 12.80	43.26 ± 13.08	0.396
**SF-36 Mental (Postoperative)**	74.90 ± 11.72	73.15 ± 14.68	73.38 ± 17.43	73.93 ± 14.39	-
**SF-36 Mental (Change, *p*-value)**	*p* = 0.001	*p* = 0.001	*p* = 0.001	*p* = 0.001	-
**VAS Lumbar Pain (Preoperative)**	7.40 ± 1.19	6.50 ± 1.73	6.46 ± 2.24	6.85 ± 1.77	0.251
**VAS Lumbar Pain (Postoperative)**	1.93 ± 1.59	2.05 ± 2.23	2.25 ± 2.00	2.07 ± 1.89	-
**VAS Lumbar Pain (Change, *p*-value)**	*p* = 0.001	*p* = 0.001	*p* = 0.001	*p* = 0.001	-
**VAS Radicular Pain (Preoperative)**	6.34 ± 1.26	6.25 ± 2.09	7.00 ± 1.25	6.53 ± 1.54	0.192
**VAS Radicular Pain (Postoperative)**	1.52 ± 1.24	1.85 ± 2.23	2.25 ± 1.87	1.85 ± 1.76	-
**VAS Radicular Pain (Change, *p*-value)**	*p* = 0.001	*p* = 0.001	*p* = 0.001	*p* = 0.001	-

**Abbreviation:** ODI = Oswestry Disability Index; RM = Roland-Morris; SF = Short Form; VAS = Visual Analog Scale;

Note:*p < 0.05 indicates statistical significance; PO = Postoperative.

**Table 3:** Comparison of Preoperative and Postoperative Radiographic Outcomes Across Surgical Techniques for Lumbar Spinal Stenosis. Table **3** presents the radiographic outcomes before and after surgery for three different surgical approaches: instrumented arthrodesis with interbody cages (Group 1), instrumented arthrodesis without interbody cages (Group 2), and uninstrumented arthrodesis (Group 3). Sagittal balance improved significantly in Groups 2 and 3 (*p* = 0.001), whereas Group 1 exhibited a smaller, non-significant change (*p* = 0.311). Lumbar lordosis remained stable across all groups, with no statistically significant postoperative changes (*p* > 0.05). These findings suggest that instrumented arthrodesis without interbody cages (Group 2) is associated with the most significant improvement in sagittal alignment, while lumbar lordosis correction is not a primary outcome of these surgical techniques.

**Variable**	**Group 1 (n=30)**	**Group 2 (n=20)**	**Group 3 (n=24)**	**Total (n=74)**	***p*-value**
**Sagittal Balance (Preoperative)**	23.37 ± 11.91	26.45 ± 13.85	33.92 ± 11.53	27.62 ± 13.00	0.668
**Sagittal Balance (Postoperative)**	20.73 ± 11.07	16.10 ± 9.33	25.04 ± 10.13	20.88 ± 10.75	-
**Sagittal Balance (Change, *p*-value)**	*p* = 0.311	*p* = 0.001	*p* = 0.001	*p* = 0.001	-
**Lumbar Lordosis (Preoperative)**	45.13 ± 8.80	46.50 ± 9.08	47.33 ± 8.52	46.22 ± 8.72	0.663
**Lumbar Lordosis (Postoperative)**	44.80 ± 6.10	48.80 ± 5.48	46.17 ± 6.12	46.32 ± 6.09	-
**Lumbar Lordosis (Change, *p*-value)**	*p* = 0.796	*p* = 0.138	*p* = 0.425	*p* = 0.894	-

**Note:**: *p < 0.05 indicates statistical significance; PO = Postoperative.

**Table 4:** Comparison of complication rates between instrumented and uninstrumented arthrodesis across studies. The table compares complication rates between instrumented and uninstrumented arthrodesis across multiple studies, highlighting significant variations. Our findings indicate a higher complication rate for uninstrumented arthrodesis (28.8%) compared to instrumented arthrodesis (8%), a trend not consistently observed in prior studies. Notably, Kaiser *et al*. (2023) and Austevoll *et al*. (2021) reported lower complication rates for uninstrumented fusion (7.73% and 5.3%, respectively). The chi-square test (*p* = 0.003) confirms a statistically significant difference between our findings and previous studies, suggesting that surgical technique, patient selection, or perioperative management may influence outcomes. While instrumentation appears to reduce some complications, rates remain variable across studies, emphasizing the need for individualized surgical decision-making based on patient-specific risk factors.

**Study**	**Instrumented (%)**	**Uninstrumented (%)**	***p*-value**
Our findings	8.0	28.8	0.003
Kaiser *et al*. (2023) [10]	14.43	7.73	
Austevoll *et al*. (2021) [4]	13.3	5.3	
Said *et al*. (2022) [15]	17.85	16.55	
Koenig *et al*. (2019) [16]	8.3	5.8	
Jalapour *et al*. (2015) [17]	6.0	5.9	

**Table 5:** Comparison of reoperation rates between instrumented and uninstrumented arthrodesis across studies. Table 5 presents a comparative analysis of reoperation rates between instrumented and uninstrumented arthrodesis across multiple studies. Our findings indicate a 12.0% reoperation rate for instrumented fusion and 8.3% for uninstrumented fusion, with a statistically significant difference compared to previous studies (*p* = 0.0003). While most studies report higher reoperation rates for uninstrumented fusion, exceptions exist, such as Jalapour *et al.* (2015), who observed a 17.9% reoperation rate for uninstrumented fusion but 0% for instrumented fusion. The variability in findings suggests that surgical technique, patient selection, and postoperative care significantly influence reoperation risks. The statistically significant difference in our findings compared to previous reports warrants further investigation into adjacent segment disease, implant failure, and long-term surgical outcomes in LSS patients.

**Study**	**Instrumented (%)**	**Uninstrumented (%)**	***p*-value**
Our findings	12.0	8.3	0.0003
Andresen *et al*. (2023) [21]	1.9	13.2	
Kaiser *et al*. (2023) [10]	11.87	14.28	
Austevoll *et al*. (2021) [4]	1.9	12.5	
Said *et al*. (2022) [15]	10.5	9.66	
Koenig *et al*. (2019) [16]	4.9	8.5	
Jalapour *et al*. (2015) [17]	0.0	17.9	

## Data Availability

The authors confirm that the data supporting the findings of this research are available within the article.

## LIST OF ABBREVIATIONS

ASD: Adjacent segment disease
BMI: Body mass index
CT: Computed tomography
IRB: Institutional Review Board
LSS: Lumbar spinal stenosis
MRI: Magnetic resonance imaging
ODI: Oswestry Disability Index
PEEK: Polyetheretherketone
RMDQ: Roland-Morris Disability Questionnaire
SDG: Sustainable Development Goals
SF-36: Short Form-36 Health Survey
SVA: Sagittal vertical axis
VAS: Visual Analog Scale

## STANDARDS OF REPORTING

STROBE guidelines were followed.
