# Supplementary material for: Surgical Outcomes of Instrumented and Uninstrumented Arthrodesis for Lumbar Spinal Stenosis: A Comparative Radiographic and Clinical Study
Source: Curr Med Imaging. 2026 Jul 14;22:E15734056415024. doi: 10.2174/0115734056415024251126104233 (PMC13728988; doi:10.2174/0115734056415024251126104233)
Supplement: Supplementary file 1 [file CMIM-22-E15734056415024_SD1.pdf]

1875-6603/26 *Send Orders for Reprints to reprints@benthamscience.net*

BENTHAM SCIENCE

CrossMark

Current Medical Imaging

Content list available at: https://benthamscience.com/journals/cmir

Surgical Outcomes of Instrumented and Uninstrumented Arthrodesis for Lumbar Spinal Stenosis: A Comparative Radiographic and Clinical Study

Carla-Lisette Garcia-Ramos[1], Julio-Roberto Perez-Oliva[1], Carlos-Alberto Carreon-Cerda[1], Diana-Laura Hernandez-Moctezuma[1], Ernesto Roldan-Valadez[2,3,*], Baron Zarate-Kalfopulos[1] and Alejandro Reyes-Sanchez[1,*]

[1]*Instituto Nacional de Rehabilitación "Luis Guillermo Ibarra Ibarra", Cirugia de Columna, 14389, Mexico City, Mexico*
[2]*Instituto Nacional de Rehabilitación "Luis Guillermo Ibarra Ibarra", Division of Research, 14389, Mexico City, Mexico*
[3]*Department of Radiology, I.M. Sechenov First Moscow State Medical University (Sechenov University), 119992, Moscow, Russia*

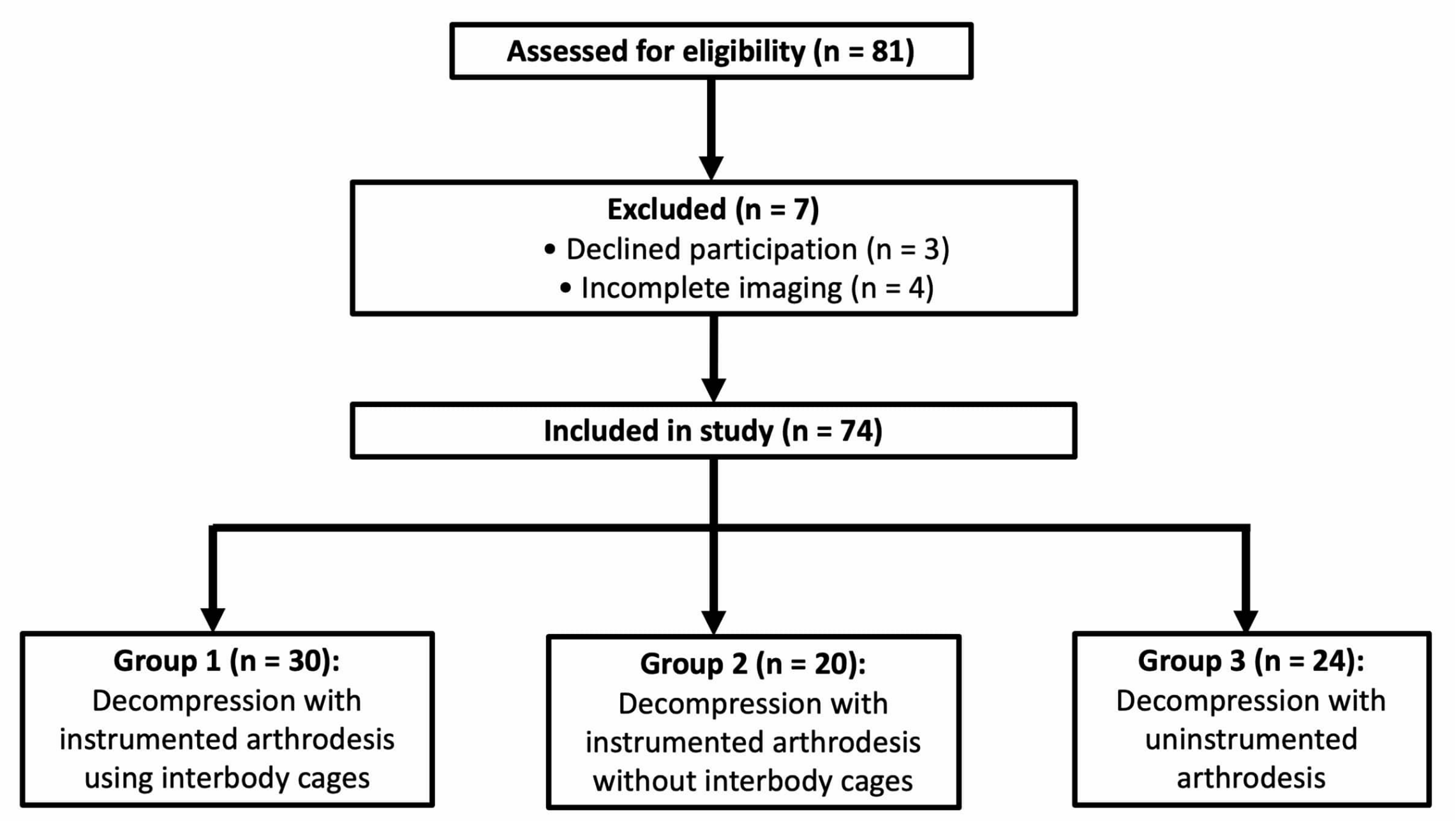

Fig. (S1). STROBE flow diagram of patient selection and group allocation.
Flowchart illustrating the recruitment and allocation of patients undergoing surgical treatment for lumbar spinal stenosis. A total of 81 patients were assessed for eligibility. After excluding 7 patients (3 declined participation and 4 had incomplete imaging), 74 patients were included in the study and stratified into three groups based on the surgical technique: Group 1 (n = 30) received decompression with instrumented arthrodesis using interbody cages; Group 2 (n = 20) received decompression with instrumented arthrodesis without interbody cages; and Group 3 (n = 24) received decompression with uninstrumented arthrodesis.

© 2026 The Author(s). Published by Bentham Science Publisher.

This is an open access article distributed under the terms of the Creative Commons Attribution 4.0 International Public License (CC-BY 4.0), a copy of which is available at: https://creativecommons.org/licenses/by/4.0/legalcode. This license permits unrestricted use, distribution, and reproduction in any medium, provided the original author and source are credited.

HOW TO CITE:
Garcia-Ramos C, Perez-Oliva J, Carreon-Cerda C, Hernandez-Moctezuma D, Roldan-Valadez E, Zarate-Kalfopulos B, Reyes-Sanchez A. Surgical Outcomes of Instrumented and Uninstrumented Arthrodesis for Lumbar Spinal Stenosis: A Comparative Radiographic and Clinical Study. Curr Med Imaging, 2026; 22: e15734056415024.
http://dx.doi.org/10.2174/0115734056415024251126104233
